# Cost‐Effective Conductive Paste for Radiofrequency Devices Using Carbon‐Based Materials

**DOI:** 10.1002/smsc.202400282

**Published:** 2024-07-22

**Authors:** Nicola Curreli, Claudia Dessì, Matteo B. Lodi, Andrea Melis, Marco Simone, Nicola Melis, Luca Pilia, Davide Guarnera, Loreto Di Donato, Alessandro Fanti, Massimiliano Grosso, Francesco Desogus

**Affiliations:** ^1^ Functional Nanosystems Italian Institute of Technology via Morego 30 16163 Genova Italy; ^2^ Transport at Nanoscale Interfaces Laboratory EMPA, Swiss Federal Laboratories for Materials Science and Technology Ueberlandstrasse 129 8600 Dübendorf Switzerland; ^3^ Clermont Auvergne INP ICCF Université Clermont Auvergne F‐63000 Clermont‐Ferrand France; ^4^ Dipartimento di Ingegneria Elettrica ed Elettronica Università degli Studi di Cagliari Via Marengo 3 09123 Cagliari Italy; ^5^ Dipartimento di Ingegneria Elettrica, Elettronica e Informatica Università di Catania Via Santa Sofia 64 95123 Catania Italy; ^6^ Dipartimento di Ingegneria Meccanica Chimica e dei Materiali Università degli Studi di Cagliari Via Marengo 2 09123 Cagliari Italy; ^7^ Dipartimento di Ingegneria dell’Informazione, dell’Infrastruttura e dell’Energia Sostenibile Università Mediterranea di Reggio Calabria Via Graziella ‐ Feo di Vito 15 89124 Reggio Calabria Italy

**Keywords:** carbon‐based pastes, high‐reactivity carbon mixtures, printed antennas, radio frequencies, screen printing

## Abstract

With the increasing demand for compact, lightweight, cost‐effective, and high‐performance radiofrequency (RF) devices, the development of low‐profile antennas becomes crucial. This article presents a study of a novel carbon–cellulose‐based paste intended for screen printing RF devices. The investigation specifically explores the application of high‐reactivity carbon mixture (HRCM) particles as conductive fillers. The results demonstrate that optimal electrical conductivity values and discrete electromagnetic dipole performances can be achieved at lower concentrations of solid conductive material compared to conventional pastes, for similar applications. This offers benefits in terms of total cost, material consumption, and environmental impact. The paste formulation showcases a complex non‐Newtonian behavior, where yielding flow and thixotropicity are found to be independent and dependent on preshear conditions, respectively. This behavior can be attributed to the network orientation and rearrangement of filler structures within the paste system, which in turn are responsible for filler pattern uniformity and overall printing quality. Compared to traditional conductive materials, HRCM pastes are proven to be a viable alternative for RF devices fabrication, including printed Wi‐Fi antennas.

## Graphical abstract

A new and cost‐effective carbon–cellulose‐based paste intended for screen printing radiofrequency (RF) devices is presented. The paste shows unique rheological properties which are well suited to fabricating Wi‐Fi dipole antennas with optimal electromagnetic response. The paste could be optimized for tailored frequencies and performance criteria of other RF devices beyond dipoles.

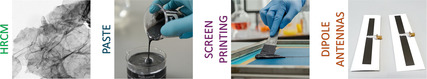

## 1 Introduction

Over time, radio tagging technology has become pervasive in everyday life, with wireless technologies like Wi‐Fi, Bluetooth, radiofrequency identification (RFID), and near‐field communication now widely adopted in various devices such as mobile phones, palm scanners, and bracelets.^[^ ^1^ ^]^ These technologies find applications in intelligent and smart packaging,^[^ ^2^, ^3^ ^]^ inventory tracking^[^ ^4^ ^]^ and tracing,^[^ ^5^ ^]^ supply chain management,^[^ ^6^ ^]^ and contactless payment.^[^ ^1^ ^]^ The global market for this technology was estimated to increase from $33.5 billion in 2022 to reach $49.7 billion by 2027, at a compound annual growth rate of 8.2% from 2022 to 2027.^[^ ^7^, ^8^ ^]^ With the growing demand for product monitoring and traceability, the use of tags for smart packaging has become crucial. These tags enable the collection and transmission of real‐time data, providing crucial information on the location and status of products along the supply chain. However, the cost of tags remains a significant barrier to wider adoption in packaging and logistics, with a target cost of 5 cents per tag for fast‐moving consumer goods.^[^ ^9^, ^10^, ^11^ ^]^ Other limitations to the widespread adoption of tags are the manufacturing methods and the materials involved. Traditional techniques for manufacturing RF antennas involve expensive and complex processes, such as lithography and chemical etching of metals. These methods require sophisticated equipment and may be suitable only for large‐scale production.^[^ ^12^ ^]^ In particular, chemical etching is a multistep process that involves the use of various pollutants, posing risks of substrate damage and restricting the range of suitable substrates for antenna implementation.^[^ ^13^ ^]^ Additionally, there is an increasing evidence of resource‐related challenges associated with the use of scarce metals such as copper, lithium, cobalt, nickel, manganese, and silver.^[^ ^14^ ^]^ These challenges include the long‐term risk of mineral depletion, the short‐term supply deficits, and the conflicts arising from limited mineral availability.^[^ ^15^ ^]^

Printed antennas with conductive pastes are emerging as a promising solution for a wide range of applications, thanks to their versatility, efficiency, and advantages over traditional manufacturing techniques. This technology offers significant potential for the production of advanced radiofrequency (RF) devices, such as RFID tags for smart packaging.^[^ ^16^ ^]^ The evolution of nanotechnology‐based materials and conductive pastes has opened up new possibilities for the fabrication of printed antennas with comparable or even superior performance to their traditional counterparts. Printing antennas with conductive pastes offers numerous advantages. First, the printing technique is more cost‐effective and scalable than standard techniques, enabling large‐scale production at reduced costs.^[^ ^17^ ^]^ Additionally, it allows for greater design flexibility, enabling the creation of antennas with customized shapes and sizes to fit various applications. Printed antennas with conductive pastes find particular utility in the field of smart packaging. Furthermore, the use of printed antennas offers the opportunity to easily integrate RFID tags into packaging materials, making the production process more efficient and sustainable.^[^ ^18^ ^]^

Various inks and pastes based on nanomaterials have been developed, including MXenes,^[^ ^19^ ^]^ semiconductors,^[^ ^20^, ^21^, ^22^ ^]^ metallic nanoparticles (MNPs),^[^ ^23^ ^]^ and carbon nanotubes (CNTs).^[^ ^24^ ^]^ However, these nanomaterials have limitations. Despite the exceptional electronic properties exhibited by MXenes, their widespread implementation in practical applications is impeded by the challenge of high production costs, necessitating ongoing research efforts to develop cost‐effective synthesis methods and enhance scalability for broader industrial adoption.^[^ ^25^ ^]^ Simultaneously, semiconductors face their own challenges, including high‐frequency performance limitations and concerns related to power consumption, highlighting the need for cost‐efficient solutions that address these drawbacks.^[^ ^26^ ^]^ Furthermore, metallic MNPs present inherent high cost and instability in common solvents, further emphasizing the need to explore economically viable and stable nanomaterial alternatives in the pursuit of advanced and cost‐effective technologies.^[^ ^25^ ^]^ Layered carbon‐based materials are promising candidates for ink and paste formulation due to their exceptional properties like chemical stability and flexibility.^[^ ^27^ ^]^ Creating printable pastes with nanomaterials is overall challenging due to the critical choice of solvent and/or particle concentration and their impact on density, surface tension, and viscosity, affecting the quality of the printing process.^[^ ^28^, ^29^ ^]^ To address this, additives like binders and thinners can tune the rheological properties for reproducible prints. Currently, high‐conductive graphene pastes for screen printing mainly involve gelation in polymer solutions^[^ ^30^ ^]^ or solvent exfoliation assisted by ethyl cellulose.^[^ ^31^ ^]^ Graphene production traditionally involves complex and costly processes, but liquid‐phase exfoliation has emerged as a more cost‐effective method.^[^ ^32^, ^33^ ^]^ However, overall costs are influenced by factors like solvent expenses, exfoliation efficiency, graphene quality, and quantity produced. Additionally, further improvement in rheological characterization of graphene‐based inks and pastes is still needed to enhance the printing process quality.

This article presents a case study on screen printing conductive pastes, utilizing low‐concentration carbon‐based active material in a nontoxic chemical solvent, a mixture of α‐terpineol and isopropyl alcohol (IPA), with ethyl cellulose (EC) as the binder. The focus is on investigating the rheological and electromagnetic properties of these pastes for RF devices. The study explores the application of high‐reactivity carbon mixture (HRCM) as active material, as previously introduced in another work with different formulations.^[^ ^34^ ^]^ In applications where constraints such as size, weight, cost, and performance dictate the need for ease of installation, the use of low‐profile antennas, such as dipoles, becomes necessary. The obtained results indicate that optimal electromagnetic response can be achieved in both static and dynamic conditions using a low‐concentration carbon‐based paste. This paste shows promising potential for realizing RF devices, including printed Wi‐Fi antennas, at significantly lower concentrations compared to conventional pastes. The paste formulation exhibits highly thixotropic behavior, characterized by its time‐dependent response, which can be attributed to the network orientation and rearrangement of filler structures within the graphene–cellulose‐based ink system. The effects of preshear and back‐and‐forth shear rate ramps further demonstrate the unique rheological properties of this paste.

## 2 Results and Discussion

### 2.1 High‐Reactivity Carbon Mixture Characterization

**Figure 1:**
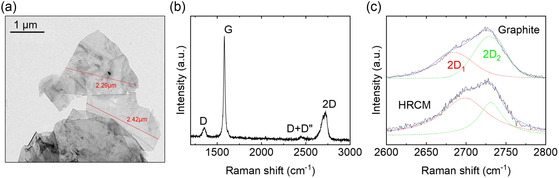
a) TEM image of the material. b) Raman spectrum of the HRCM material. c) Detail of the Raman 2D peak of the material compared to that of graphite, including a study of the deconvolution of the 2D peak into two components 2D_1_ and 2D_2_.

The lateral size of the HRCM material was analyzed using transmission electron microscopy (TEM, **Figure** **1**a and S1, Supporting Information). The TEM analysis revealed the presence of agglomerates of carbon material, with some of these agglomerates exhibiting a flake‐like morphology with a high aspect ratio (average lateral size of 2.3 μm). In addition to TEM, the structural properties of the as‐produced HRCM material were also characterized using Raman spectroscopy (Figure 1b and S2, Supporting Information). We observed a mode at $\approx 1580\;{\text{cm}}^{-1}$, which corresponds to the high‐frequency E_2g_ Raman‐allowed optical phonon. Additionally, a second peak at ≈1350 cm^−1^ was detected, and this peak was assigned to an A_1g_ breathing mode. The presence of this mode is commonly observed in defected and nanocrystalline graphite.^[^ ^35^ ^]^ Similar bands have been observed in various polyaromatic hydrocarbons.^[^ ^35^ ^]^ For historical reasons, in carbon allotropes, the E_2g_ mode is commonly referred to as the G peak, derived from ‘graphite’, as it exhibits a strong intensity in pristine graphite. Conversely, the A_1g_ mode is known as the D peak, derived from ‘disorder’, as it appears predominantly in defected graphite. In the case of the investigated HRCM, being composed of carbon atoms, the assignment of these modes becomes straightforward. The higher‐frequency band corresponds to the bond stretching of sp^2^ pairs present in both rings and chains, whereas the lower‐frequency band corresponds to radial breathing modes of sp^2^ atoms within the rings. Consequently, in the absence of rings, the lower‐frequency band would be absent, while the higher‐frequency band is present in all carbon materials, ranging from carbon chains to hard amorphous carbons. This suggests that in the case of HRCM, the presence of the peak at ≈1350 cm^−1^ indicates the affinity of HRCM to other materials containing sp^2^ atoms in rings, such as graphene. Furthermore, two additional peaks have been observed in the spectrum: one located at ≈2700 cm^−1^ and a weaker one at ≈2450 cm^−1^. In pristine graphite, these peaks are attributed to overtone vibrations and are assigned as 2D and D + D″, respectively. Based on the observed Raman signature, it is possible to conclude that HRCM exhibits a Raman spectrum typical of carbonaceous materials. However, it differs from graphite due to the presence of the D peak, which is not observed in pristine graphite but is more similar to the signature of materials possessing a ring‐shaped structure, such as graphene. Additionally, the 2D peak is a prominent feature in the Raman spectrum of carbon materials and it can be analyzed by deconvoluting it into multiple components, the primary ones being 2D_1_ and 2D_2_.^[^ ^35^ ^]^ The intensities of these components are roughly 1/4 and 1/2 of the intensity of the G peak (Figure 1c).^[^ ^35^ ^]^ It is possible to observe more complex 2D peak shapes than those observed in Bernal stacked materials.^[^ ^35^ ^]^ In principle, any relative orientation and stacking of the layered structure could be possible, and this would be reflected in a significant change of the band structure of HRCMs. Thus, a detailed study of the 2D peak shape can bear important information on the multilayer interactions. By evaluating the intensity ratio of the two main components of the 2D band (i.e., I(2D_1_) and I(2D_2_)) it is possible to estimate the flake thickness.^[^ ^35^ ^]^ Considering a ratio defined as

(1) $$I(2D_{1})/I(2D_{2})=\alpha_{\text{2D}}$$

samples whose $\alpha_{\text{2D}}>1$ can be assumed to be <10 nm thick; on the other hand, the ones whose $\alpha_{\text{2D}}<1$ are considered >10 nm thick, being electronically indistinguishable from graphite.^[^ ^36^ ^]^ In the case of HRCM we found a $\alpha_{\text{2D}}=1.15$; hence, the it is possible to estimate the average thickness of HRCM <10 nm. The preparation details for the HRCM and the characterization methods can be found in the Supporting Information of this article.

### 2.2 Thermogravimetric Analysis

**Figure 2:**
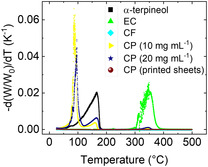
First‐derivative (DTG) curve illustrating the percentage weight change of the sample with respect to temperature in TGA tests. The data points correspond to α‐terpineol (black squares), ethyl cellulose (EC, green triangles), carbon‐based filler (CF, cyan rhombi), conductive paste (CP) at $10\;\text{mg}\;{\text{mL}}^{-1}$(yellow triangles) and $20\;\text{mg}\;{\text{mL}}^{-1}$ (blue stars). Dark red dots represent the DTG curve for the conductive paste of the printed sheets after annealing.

**Figure** **2** shows the thermogravimetric analysis results on the HRCM‐based conductive paste and its components in terms of the first derivative of the sample percentage weight with respect to the temperature derivative thermogravimetry (DTG curve). The comparison between the outputs for the conductive pastes and the components allows a deeper understanding of the thermal response of the paste, an estimation of its composition after each treatment step, and an explanation of the printed sheet processing. The fresh paste, at the initial concentration of 10 $\text{mg}\;{\text{mL}}^{-1}$, undergoes the most consistent weight loss with a maximum rate at about 87 °C, at which the weight is 65.0% of the initial sample mass, and stabilizes at about 118 °C, which corresponds to a residual weight of about 16.4%. A second weight loss is encountered with a maximum rate near 161–162 °C, corresponding to 6.8% of the initial weight, and completes at about 192 °C, at which 4.8% of the initial mass is still present. The latter is a good estimation of the filler and binder weight contribution, as at this temperature, IPA has completely evaporated. On the other hand, α‐terpineol has almost completed evaporating: indeed, α‐terpineol alone presents a maximum weight decreasing rate at about 166–167 °C (the sample mass is 16.3% of the initial one) and, at 202 °C, only 0.5% of the initial mass is still present (the boiling temperature of α‐terpineol is about 219 °C at the atmospheric pressure^[^ ^37^ ^]^). The fresh paste's third (and last) main weight loss happens with a maximum rate at 344 °C, corresponding to 3.9% of the initial weight, and finishes at about 372 °C, for which a residual weight of 3.3% is registered. This phase is due to binder degradation and loss. Indeed, EC alone presents a first slight weight loss with the maximum rate and quick completion between 225 and 227 °C, corresponding to 98.8% of the initial weight, a second very small loss with its maximum rate at about 299–30 °C, corresponding to 97.8% of the initial mass, and completion at about 302 °C, when a residual weight of 97.6% is registered, a main weight loss with two near maximum peaks at about 347–348 °C (where the sample mass reaches 48.5% of the initial one) and 354–355 °C (34.1% of the initial mass is reached), which is almost completed at about 49 °C, where a residual mass of 7.3% is still present. If the maximum weight loss rates are compared for the conductive paste and EC alone in the range 344–348 °C, the contribution of about 2.8% for EC alone on the total paste mass can be estimated. It should also be noted that the solvent content in the mixture is 95.2%. Among this, 18.1% constitutes α‐terpineol, calculated by comparing the maximum weight loss rate of the paste and of α‐terpineol in the range 161–167 °C, and 77.1% is accounted for by IPA. The remaining 4.8% is made up of filler and binder components. These values are near the theoretical ones in the paste formulation, 19.0% and 79.8% for α‐terpineol and IPA, respectively. For the paste concentrated at $20\;\text{mg}\;{\text{mL}}^{-1}$, comparing the maximum weight loss rates for the conductive paste and EC alone in the range 346–348 °C, an EC mass of 4.7% on the total paste mass can be estimated. Considering the carbon filler mass to be constant, 92.5% of the solvent is still present, made of 31.5% of α‐terpineol, calculated by comparing the maximum weight loss rate of the paste and of α‐terpineol in the range 165–167 °C, and the remaining 61.0% of α‐terpineol. In the case of the printed sheets, the analysis of the mass‐loss rate reveals that no significant changes happen at temperatures below 325 °C, near which a very small and negligible decrease occurs. This demonstrates that no solvent is still present in the sheets and that the annealing treatment results in a permanent stabilization of the printed material composition. By assuming the carbon filler mass is constant, a final EC content of 4.2% can be estimated. The thermal behavior of ethyl cellulose can be explained by different reaction processes.^[^ ^38^, ^39^ ^]^ At first, dehydration occurs with crosslinking of cellulose chains. Compared to pure cellulose, EC does not decompose via transglycosylation reaction and the formation of levoglucosan due to the presence of ethylated substituents, which, in turn, induces the formation of compounds that often are the corresponding ethylated form of cellulose pyrolysis products.^[^ ^39^ ^]^ However, up to 27 °C, the basic structure of the cellulose chain is overall retained, even if interchain ether formation is possible, which results in a 3D structure with more stable ether bonds, which can finally bring to char formation.^[^ ^39^ ^]^ At higher temperatures, the fragments further decompose, producing volatile compounds and tars.^[^ ^39^ ^]^

### 2.3 Steady Shear Rheology

**Figure 3:**
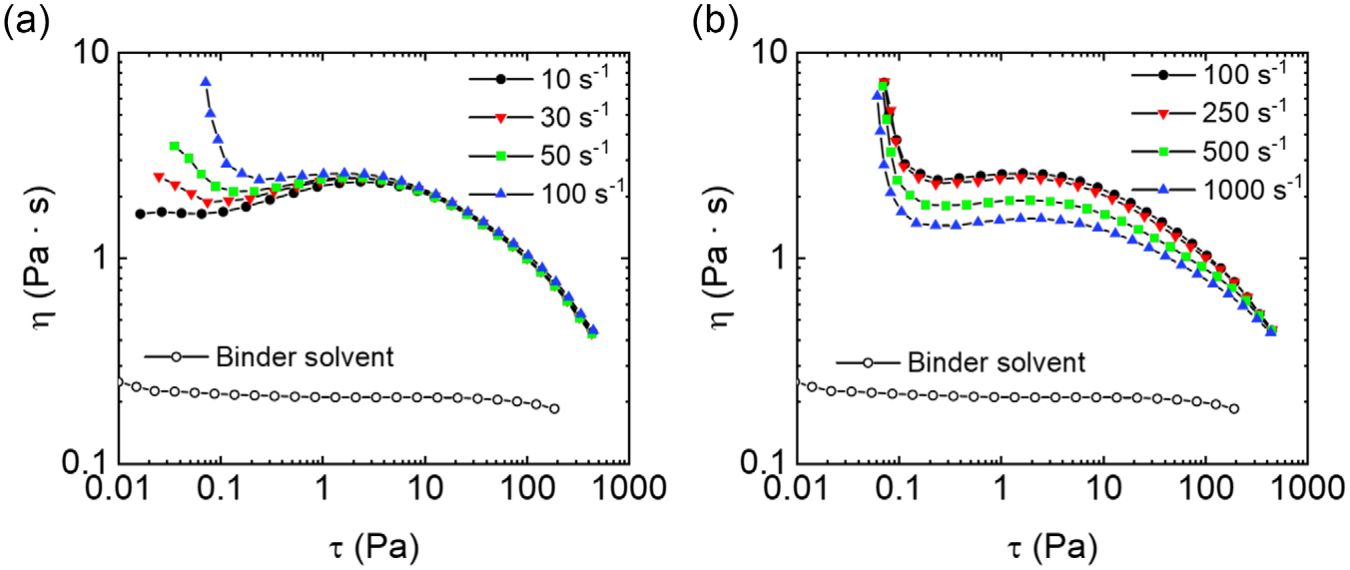
Shear viscosity from flow curve measurements as a function of shear stress is reported for the conductive paste with EC:filler ratio 1:1 with respect to EC/solvent mixture at 2 °C where different preshear values are applied for 2 min: a) from 10 to 100 s^−1^ and b) from 100 to 1000 s^−1^.

In order to obtain a printing paste suitable for tape casting or screen printing, a careful tuning of rheological parameters in shear flow should be considered, taking into account the operation specifications of these printing techniques as well.^[^ ^40^, ^41^ ^]^ In this regard, the effect of applied preshear deformation on the characteristics of yielding and thixotropicity observed for the printing paste is investigated at ambience temperature. Preshear deformations, yielding, and thixotropicity are, indeed, flow conditions often related to the quality of printed films. In particular, preshear deformations may describe the past flow history of a paste in case the latter has to be preprocessed right before printing, the presence of yielding may induce sedimentation issues at the beginning of the printing process, whereas a thixotropic behavior may be responsible of less precise printed film edges. **Figure** **3** shows shear viscosity (*η*) obtained from flow curve measurements for a conductive paste concentrated at 20 $\text{mg}\;{\text{mL}}^{-1}$, with an EC/HRCM particle ratio of 1:1, alongside the viscosity of the EC/solvent mixture at 2 °C. In particular, the shear viscosity (*η*) as a function of shear stress (*τ*) is reported after applying preshear values from 10 to 100 s^−1^ (Figure 3a), and from 100 to 1000 s^−1^ (Figure 3b). The maximum value of applied shear rate, 1000 s^−1^, represents the flow conditions typical of screen printing techniques using mesh tools.^[^ ^41^ ^]^

Compared to EC/solvent mixture, which shows Newtonian flow behavior up to 1000 s^−1^, an apparent non‐Newtonian flow response is depicted for the conductive paste. Such non‐Newtonian flow varies according to the applied preshear value. In Figure 3a, from 10 to 100 s^−1^ preshear, a transition from Newtonian to shear‐yielding flow behavior is observed at low stresses (from 0.02 to 0.2 Pa), followed by a transition from pseudoplastic to quasi‐Newtonian response at midstresses (from 0.3 to 5 Pa). Eventually, a shear‐thinning flow independent of the applied preshear (i.e., expected same shear‐thinning index) occurs. In Figure 3b, upon further increase of the applied preshear value from 100 to 1000 s^−1^, both yielding and shear‐thinning flow regions at low and high stress, respectively, separated by a quasi‐Newtonian region at intermediate stress values, are observed. This is a complex non‐Newtonian flow behavior generally ascribed to anisotropic systems, for example, liquid crystal polymers^[^ ^42^ ^]^ and 2D plate‐like particle suspensions.^[^ ^43^ ^]^ For printing pastes, the presence of a second Newtonian region often represents the optimal printing window in terms of applied shear load or deformation where the quasiconstant values of the shear viscosity ensure uniform thickness of the printed film. In our case, the interval of the second Newtonian region depends on the applied preshear deformation, that is, the higher the preshear value, the wider the second Newtonian region. Moreover, increasing the preshear value induces a decrease of the viscosity curve in the whole stress interval and the lower shear‐thinning response at high stress. If yield stress is approximately defined as the value of the stress at the maximum viscosity only when the latter is followed by an abrupt decrease, it is worth to note that such yield stress is observed only for conductive pastes subjected to preshear of 100 s^−1^ and higher, showing a similar value for all the applied preshear values (i.e., ≈0.07 Pa). Since in this work the assessed binder concentration is 2.3–2.4 wt%, EC is not expected to aggregate in *α*‐terpineol‐based solutions in the first place.^[^ ^41^ ^]^ This may induce binder chains to act most likely as a dispersant agent for the conductive filler in the paste (i.e., less friction between binder chains and filler particles), while promoting better particle wetting with solvent molecules as well. However, when a preshear deformation is applied to such heterogeneous systems, an apparent, but also temporary, disordered/aggregate state of the particles seems to induced, to which the appearance of pseudoplasticity and yielding could be related. In our case, due to very high filler aspect ratio, HRCM particles may form a structural network in the first place, whose order level depends on the particle orientation, which in turn depends on subsequent applied deformation field as well. The ultimate shear‐thinning flow behavior for filled systems often indicates the presence of a weakly attractive network among filler particles. Upon increasing the applied shear rate, the filler–filler network tends to break down, following the progressive alignment of polymer chains of the binder under the imposed shear deformation flow. However, regardless the initial disordered particle state, the beginning of the ultimate shear‐thinning flow is observed to occur at the same stress value developed within the system (i.e., ≈6 Pa). This might affect the capability of printed film edges to avoid further material flow once the printing process is terminated, altering the precision of the printing quality.

**Figure 4:**
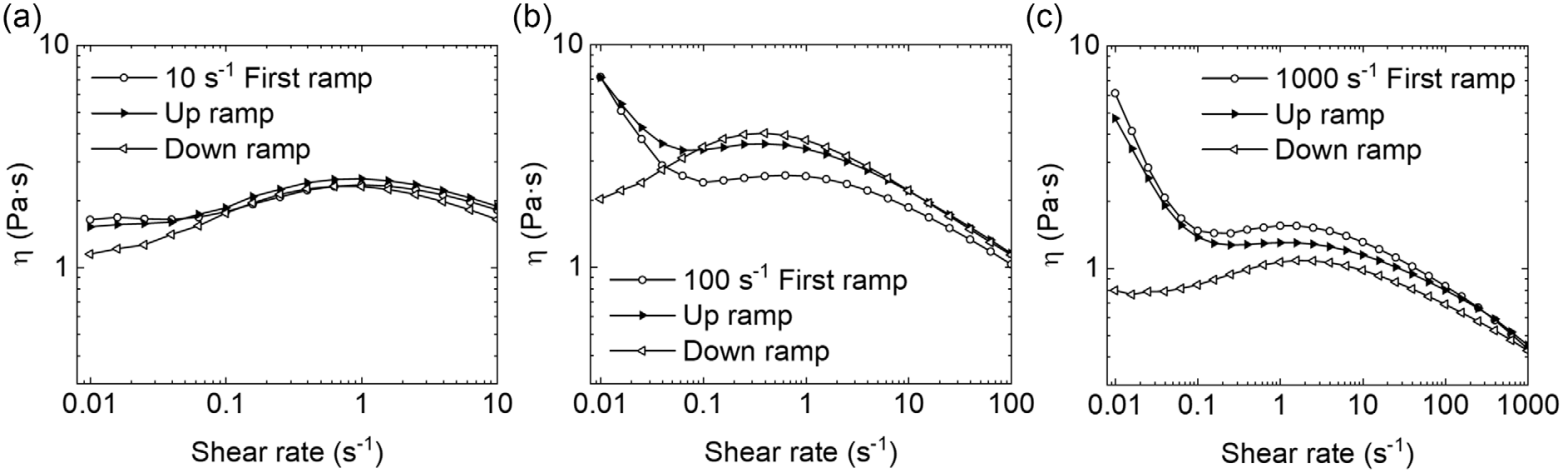
Up and down shear viscosity ramps for the conductive paste with EC:filler ratio 1:1 at 2 °C after the initial flow curve where different preshear values were applied for 2 min: a) 10 s^−1^, b) 100 s^−1^, and c) 1000 s^−1^.

The presence of thixotropicity and the orientation degree of the filler structural network within our self‐prepared pastes is assessed by performing up and down shear rate ramps right after the first flow curve with applied preshear values of 10, 100, and 1000 s^−1^ (**Figure** **4**a–c, respectively). Both up and down shear rate ramps never overlap the starting flow curve entirely, which is a general sign of thixotropic behavior. At applied preshear of 10 s^−1^, they show a flow behavior similar to the initial flow curve, which weakly depends on the applied shear rate, although viscosity tends to slightly lower values for the down shear rate ramp from 0.1 s^−1^ and lower (Figure 4a). Indeed, low‐enough preshear values are not capable to induce a specific ordering direction either to polymer chains of the binder or to filler particles. However, a slightly higher disordered structure of filler particles may lead to an increased number of filler–filler contacts and, thus, higher friction phenomena between filler particles. In the case of preshear values of 100 and 1000 s^−1^, on the other hand, up and down shear rate ramps show a different behavior between each other (Figure 4b,c, respectively). In particular, up shear rate ramps resemble the shear flow behavior of the initial flow curve, whereas down shear rate ramps show an apparent departure from the initial flow curve, especially in the mid–low‐rate range. The observed phenomenon can be explained by stating that, for filled systems, the successive flow deformations may give enough time to the filler network to rearrange itself to almost its initial condition (up shear rate ramps) or may not (down shear rate ramps). Moreover, at the intermediate preshear value of 100 s^−1^, the progressive loss of orientation degree of filler particles upon decreasing the applied shear rate leads to higher friction between particles starting from 5 s^−1^, showing a reversed shear‐thickening flow behavior at lower rates, in place of the quasi‐Newtonian and yielding flow regions of the original flow curve (Figure 4b). This is qualitatively observed for the highest preshear case (1000 s^−1^) as well (Figure 4c). However, the down shear rate ramp shows viscosity values much lower than the initial flow curve, creating hysteresis‐like flow. In this case, a permanent structural change has occurred within the conductive paste over time, and most likely any initial filler–filler structure cannot longer be recovered. The formulation details for the printable pastes can be found in the Supporting Information of this article.

### 2.4 Dipole Design and Characterization

We investigated the suitability of HRCM materials for RF applications, such as the design and fabrication of efficient dipole antennas. To verify the effectiveness of HRCM as material to implement electromagnetic devices in microwave range, we designed, manufactured, and tested a printed dipole. In this prototype, the HRMC‐based paste is deposited on a ≈0.5 mm‐thick cardboard substrate.

First, we modeled a half‐wavelength dipole antenna based on the fundamental antenna theory.^[^ ^44^ ^]^ Dipole antenna is commonly used to test the suitability of new materials due to its simplicity and its suitability to be realized via printed technologies.^[^ ^45^, ^46^, ^47^ ^]^ Basically, a half‐wavelength dipole consists of two quarter‐wavelength conductors *ℓ* long, called arms, placed end to end for a total antenna length of $L=2\ell =\frac{\lambda }{2}$. If such an antenna is fed via an electric field in the small gap between the two conductors, an electric current is induced on its surface. Such a current in turn produces an electric field, which is the electric field radiated by the antenna. The dipole length is calculated as follows:^[^ ^44^ ^]^

(2) $$L=\frac{468\times 30.48}{f}$$

where *L* is expressed in cm and *f* is the desired frequency in MHz. The electric dipole can be arranged as a wire or as a printed antenna.^[^ ^44^ ^]^

Subsequently, a full‐wave analysis has been carried out using the software CST Studio Suite, based on the finite integration technique (Time Domain Solver) in order to fine tune the model of the HRCM‐based half‐wave printed dipole antenna. This software allowed us to further optimize the antenna and analyze its electromagnetic performance and verify the effective operation before the realization. The simulation details can be found in the Supporting Information of this paper.

**Figure 5:**
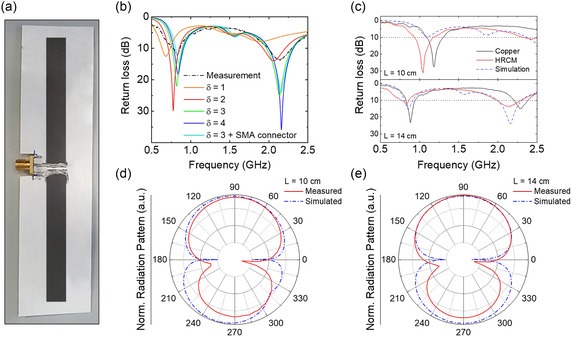
a) Photograph of a dipole antenna implemented by screen printing using HRCM material. b) Simulated return loss of the HRCM‐based dipole antenna in order to evaluate the electrical conductivity of the HRCM material. c) Measured return loss of the 10 cm (top) and 14 cm (bottom) dipole antennas made with copper (black curve) and HRCM (red curve). The simulated return loss of the HRCM antenna is provided for comparison (dashed blue curve). d) Normalized radiation pattern of the 10 cm HRCM dipole antenna. e) Normalized radiation pattern of the 14 cm HRCM dipole antenna. The red curves represent the measured radiation pattern of the realized HRCM antenna, while the blue dashed curves represent the radiation pattern of the simulated HRCM‐based antenna.

To explore the versatility of HRCM across different frequency bands, we designed two dipoles operating at ≈1 and 1.4 GHz, respectively. These frequencies are of particular interest due to their relevance in several ultrahigh‐frequency applications (e.g., RFID, communications, etc.). For our initial design, we selected the conductivity of copper ($5.96\times 10^{7}\;{\text{Sm}}^{-1}$ @ 2 °C), which is a widely used material for antenna fabrication, as a reference for evaluating the performance of HRCM. In order to assess the suitability of HRCM as an alternative material for antenna design in these frequency ranges, we carried out simulations using CST Studio Suite software to determine the electrical conductivity of the HRCM material. Through iterative simulations, we systematically tuned the conductivity of the HRCM material defined in the software, aiming to identify the conductivity value that minimizes the difference between the simulated antenna characteristics and the corresponding experimental measurements. Hence, we implemented two sets of prototypes: one made of copper and another made of HRCM, both with a length of L= 14 cm (corresponding to half‐dipole lengths of ℓ=7 cm) and a width of 1 cm. The two half dipoles were spaced 3 mm apart. The copper dipole antenna was fabricated by metalizing a cardboard foil using adhesive copper tape (copper thickness 35 μm). On the other hand, HRCM dipole antenna was fabricated using screen printing on a cardboard foil and directly connected to an SubMiniature version A (SMA) connector soldered with silver paste (**Figure** **5**a). The fabrication details for the dipoles can be found in the Supporting Information of this article. The resulting HRCM dipoles demonstrated remarkable flexibility, allowing for random twisting. Additionally, the HRCM dipoles exhibited lower weight and thickness compared to their copper counterparts. Subsequently, we measured the average resistivity of the printed HRCM dipole antenna, obtaining a value of (250.0 ± 0.6) Ω cm. The typical dry print thickness was determined to be (305 ± 6) μm, resulting in a sheet resistance of ≈50 Ω sq^−1^. These values served as the starting point for the simulations of electrical conductivity. Following the resistivity measurements, we proceeded to measure the scattering parameters of both the copper and HRCM dipole antennas to gain insights into their impedance matching and radiation characteristics. These parameters are best described and measured by the cross‐coupling coefficients (S_mn_) of the antenna scattering matrix. Specifically, the return loss (−S_11_) quantifies the power reflected back to the source by the antenna.^[^ ^44^ ^]^ By definition, transmitted power is not reflected back, so measuring the S_11_ allows us to determine (net of power losses) the transmission band of the printed antenna.

Scattering parameters were acquired using a vector network analyzer and coaxial cables were connected to the SMA connector for the measurements. For the purpose of comparative analysis, we conducted simulations using CST, where we designed and simulated a dipole antenna with dimensions identical to those of the reference copper dipole. To do this, we introduced a parameter, denoted as *δ*, to tune the electrical conductivity of copper ($5.96\times 10^{\delta }\;{\text{Sm}}^{-1}$). By systematically varying *δ* and analyzing the simulated results, we aimed to identify the value of *δ* that would best match with the behavior of measured HRCM‐based antennas. After multiple simulations with different values of *δ*, we determined that δ=3 provided the closest match to HRCM's behavior, resulting in an effective conductivity of $\approx 6\times 10^{3}\;{\text{Sm}}^{-1}$ for HRCM. This conductivity value was four orders of magnitude lower than that of copper, as illustrated in Figure 5b. Subsequently, we extended our simulations to assess the performance of the dipole antenna with HRCM in a real‐world scenario, considering the perturbations introduced by the presence of an SMA coaxial cable. Through these simulations, we determined the actual return loss of the antenna under practical conditions. After determining the electrical conductivity of the HRCM, we proceeded to evaluate the performance of dipole antennas based on HRCM for transmitting and receiving RF electromagnetic signals. To assess their capabilities, we measured both the return loss and the radiation pattern of the two dipole antennas. The dipole antennas were simulated with a total length of L=10 cm and L= 14 cm, which corresponded to half‐dipole lengths of ℓ=5 cm and ℓ=7 cm, respectively, and a thickness of ≈300 μm. The width of each dipole was 1 cm. The two half‐dipoles were spaced 3 mm apart (Figure 5a). Coaxial cables were connected to the SMA connector for the measurements. For comparison, a reference dipole with identical geometry but made of copper was also fabricated. The reference dipole had a thickness of 35 μm, which is commonly used in commercial PCBs and printed flexible circuits. The electromagnetic characterization results of the HRCM paste‐based dipole antennas are provided in Figure 5c. In reference to the 10 cm‐long antenna, a copper antenna is expected to exhibit resonance at a specific frequency of λ/2 = 1.4265 GHz. However, in our investigation of the dipole implemented with HRCM, we found that it resonates at a lower frequency of 1.0421 GHz, covering a −10 dB bandwidth of 240 MHz (from 935 to 1174 MHz). Conversely, the realized copper dipole antenna resonates at a frequency of 1.2 GHz, which is reasonably close to the theoretically expected value of 1.4265 GHz, covering a −10 dB bandwidth of 210 MHz (from 1.095 to 1.305 GHz). This proximity suggests that the copper antenna performs as anticipated based on theoretical calculations and design considerations. The higher conductivity of copper compared to HRCM enables more efficient signal transmission and reception, resulting in a resonant frequency closer to the theoretical prediction. It can be observed that the conductive HRCM‐based paste resonates at a frequency ≈150 MHz lower than the copper dipole with same dimensions. This indicates that the equivalent electrical length is slightly higher for the HRCM‐based paste. Furthermore, there is a ≈30 MHz difference in the −10 dB bandwidth between the HRCM‐based paste and copper material. The larger bandwidth suggests that the synthesized material is not an ideal conductor, but rather a lossy metal with finite conductivity. In fact, HRCM, being a mix of carbon‐based materials, possesses different electrical properties compared to copper, particularly a lower conductivity, which affects the propagation of electromagnetic waves and leads to a shift in the resonant frequency. The results obtained from our simulation analysis are consistent with the experimental findings, providing further validation for the accuracy of the simulation model. This agreement strengthens the reliability of our results. As the dipole size increases, the working frequencies decreases. Moving to the 14 cm‐long antenna, a copper antenna is expected to exhibit resonance at a specific frequency of 1.019 GHz. However, in our investigation of the dipole antenna fabricated with HRCM, we found that it resonates at a lower frequency of 832 MHz, with a −10 dB bandwidth of 108 MHz (from 762 to 870 MHz). In comparison, the realized copper dipole antenna resonates at a frequency of 885 MHz with a −10 dB bandwidth of 126 MHz (from 825 to 951 MHz). The simulation analysis aligns with the experimental findings. Here, the difference in resonance frequency between the copper and HRCM‐based samples reduces to 187 MHz, while the bandwidth is ≈20 MHz lower (Figure 5c). There is a better agreement between the simulation and experimental data for this case, with the best input impedance matching (Figure 5c). However, it should be noted that a decrease in the working bandwidth is observed, suggesting that the conductivity of the HRCM paste is not constant across frequency, and the losses may decrease as the frequency decreases. Moreover, it is worth mentioning that a second resonance, occurring almost at 2.5 times the resonance frequency, becomes more prominent as the resonance frequency decreases (i.e., as the dipole size increases). The observed difference in resonant frequencies between the copper and HRCM antennas can be attributed to the dissimilar conductivity characteristics of the two materials. HRCM, as a composite material, possesses different electrical properties compared to copper. Its lower conductivity affects the propagation of electromagnetic waves, resulting in a shift in the resonant frequency. HRCM's inferior conductivity relative to copper leads to a lower resonant frequency. However, despite this shift, the HRCM antenna still demonstrates a desirable bandwidth, making it suitable for certain applications where a broader frequency range is more important than a precise resonance. The broad bandwidth exhibited by the HRCM antenna suggests its effectiveness in transmitting and receiving signals within this frequency range, making it suitable for various communication applications. This supports the use of HRCM as a viable alternative material for antenna manufacturing, especially when compared to expensive materials like copper. The radiation pattern analysis of HRCM dipole antennas is given in **Figure** **5**d,e. The radiation pattern exhibited a similar toroidal shape to that of a conventional dipole antenna, the slight asymmetry is due to the coaxial power supply. However, due to the different electrical properties of the HRCM material compared to traditional conductors, there were notable variations in the radiation characteristics. In terms of the bidimensional radiation pattern, the HRCM dipole antenna demonstrated a slightly broader distribution of energy compared to a conventional copper dipole antenna. The main lobe, representing the direction of maximum radiation, exhibited a wider beamwidth in both the horizontal and vertical planes. This broader main lobe indicates a wider coverage area for the HRCM dipole antenna. Additionally, the HRCM dipole antenna displayed reduced sidelobes compared to the conventional dipole. The energy radiation in directions perpendicular to the main lobe was significantly attenuated. This characteristic suggests a more focused radiation pattern in the desired direction of maximum radiation. Simulated radiation patterns were obtained for HRCM dipoles, demonstrating agreement with conventional copper‐based dipole structures (Figure 5d,e).

## 3 Conclusion

In this study, we investigated a new conductive paste formulation based on ethyl cellulose and filled with HRCM nanoparticles for its application in screen‐printed RF devices. The HRCM particles exhibited a characteristic Raman spectrum typical of carbon‐based materials and showed a high aspect ratio, similar to that found in graphene. Shear rheological characterization revealed a complex non‐Newtonian fluid response of the HRCM‐based paste compared to the unfilled control system. This behavior displayed an alternation of flow properties depending on the applied shear rate and preshear values. In particular, mechanical properties such as yielding flow and thixotropicity were evaluated, highlighting the presence of a particle network and its rearrangement under shear deformation flow. These findings provide insights into optimizing the flow properties of the paste, enabling the control of printing performance during screen printing application processes.

Subsequently, to demonstrate the applicability of HRCM‐based pastes, we implemented proof‐of‐concept dipole antennas. The resulting HRCM‐based dipole antennas exhibited a broader bandwidth than copper antennas, demonstrating the dissipative nature of the HRCM‐based paste. The radiation patterns displayed broader bands compared to copper antennas, resulting in wider beamwidths in both horizontal and vertical planes, enhancing signal coverage. This feature could be advantageous in scenarios requiring robust signal propagation, such as wireless communication networks, IoT devices, satellite communications, and radar systems operating in diverse environmental conditions.

Due to their lower production cost and enhanced mechanical flexibility, HRCM‐based pastes could serve as a viable alternative material for implementing RF devices, particularly in applications where broader bandwidth is critical. Perspectively, these pastes could be optimized for specific frequency ranges and performance criteria to be used in different types of antennas beyond dipoles, thereby expanding their applicability in RF technology.

## Conflict of Interest

The authors declare no conflict of interest.

## Author Contributions

**Nicola** **Curreli**: Conceptualization: (equal); Data curation: (lead); Formal analysis: (equal); Funding acquisition: (equal); Investigation: (equal); Methodology: (equal); Project administration: (equal); Resources: (equal); Supervision: (equal); Visualization: (lead); Writing—original draft: (equal); Writing—review and editing: (equal). **Claudia** **Dessì**: Conceptualization: (equal); Data curation: (lead); Formal analysis: (equal); Investigation: (lead); Methodology: (equal); Validation: (lead); Visualization: (lead); Writing—original draft: (equal); Writing—review and editing: (equal). **Matteo** **Bruno** **Lodi**: Conceptualization: (equal); Data curation: (equal); Formal analysis: (equal); Investigation: (lead); Methodology: (equal); Software: (equal); Visualization: (equal); Writing—original draft: (equal); Writing—review and editing: (equal). **Andrea** **Melis**: Data curation: (equal); Investigation: (lead); Software: (equal). **Marco** **Simone**: Formal analysis: (equal); Validation: (equal); Visualization: (equal); Writing—review and editing: (equal). **Nicola** **Melis**: Data curation: (equal); Investigation: (equal); Validation: (equal); Writing—review and editing: (equal). **Luca** **Pilia**: Conceptualization: (equal); Data curation: (equal); Investigation: (equal); Methodology: (equal); Resources: (equal); Supervision: (equal); Writing—original draft: (equal); Writing—review and editing: (equal). **Davide** **Guarnera**: Data curation: (equal); Investigation: (lead). **Loreto** **Di** **Donato**: Data curation: (equal); Investigation: (equal); Resources: (equal); Supervision: (equal). **Alessandro** **Fanti**: Conceptualization: (equal); Funding acquisition: (equal); Investigation: (equal); Project administration: (equal); Resources: (equal); Supervision: (equal); Writing—review and editing: (equal). **Massimiliano** **Grosso**: Conceptualization: (equal); Data curation: (equal); Formal analysis: (equal); Investigation: (equal); Methodology: (equal); Resources: (equal); Supervision: (equal); Validation: (lead); Visualization: (equal); Writing—original draft: (equal); Writing—review and editing: (equal). **Francesco** **Desogus**: Conceptualization: (equal); Data curation: (equal); Formal analysis: (equal); Funding acquisition: (equal); Investigation: (equal); Methodology: (equal); Project administration: (equal); Resources: (equal); Supervision: (equal); Visualization: (equal); Writing—original draft: (equal); Writing—review and editing: (equal).

## Supporting information

Supplementary Material

## Data Availability

The data that support the findings of this study are available in the supporting information of this article.
