# Supplementary material for: Cost‐Effective Conductive Paste for Radiofrequency Devices Using Carbon‐Based Materials
Source: Small Sci. 2024 Jul 22;4(11):2400282. doi: 10.1002/smsc.202400282 (PMC11935040; doi:10.1002/smsc.202400282)
Supplement: Supplementary file 1 — Supplementary Material [file SMSC-4-2400282-s001.pdf]

Supporting Information

Cost-effective Conductive Paste for Radio-frequency Devices using Carbon-based Materials

Nicola Curreli[†,*], Claudia Dessì[†], Matteo B. Lodi, Andrea Melis, Marco Simone, Nicola Melis, Luca Pilia, Davide Guarnera, Loreto Di Donato, Alessandro Fanti*, Massimiliano Grosso, Francesco Desogus*

1. Experimental Section

a. High Reactivity Carbon Mixture (HRCM)

We prepared a dispersion of HRCM using the formulation strategy described in the work by Hyun and colleagues. [1] HRCM, used as acquired, comes from the production process outlined in the patent US7842271B2, which involves a "cold destruction reaction". This process induces the expansion of graphite layers through an autocatalytic chain reaction.

b. Formulation of Printable Pastes

Conductive pastes are made of three main components:

i) conductive solid phase of filler particles;

ii) binder which stabilizes and/or functionalizes the filler particles;

iii) liquid phase called solvent medium where filler particles are dispersed together with the binder.

The conductive solid phase is HRCM material, the binder is ethyl cellulose (with a viscosity of 22 mPa s in 80:20 toluene/ethanol, Sigma Aldrich), and the solvent medium is a mixture of α-terpineol (90 % purity, average molecular weight M_w = 154.25 g mol^{-1}, boiling temperature T_b = 217–218°C, Sigma Aldrich) and isopropyl alcohol (Sigma Aldrich).

For the preparation of the conductive paste, filler particles and binder with both concentrations of 10 mg mL^{-1} were embedded to the solvent medium after being weighed on a precise small-scale balance. The filler:binder weight ratio is 1:1 and kept constant, whereas the solvent medium is a mixture of IPA:α-terpineol with volume ratio 5:1 following the paste formulation proposed by Hyun et al. in their work. [1] The dispersion of filler particles and binder into the solvent medium was achieved by degassing sonication for 4 h in an ultrasound bath (Xmoonant Ultrasonic Cleaner, 40 kHz, 35 W). The resulting suspension was then concentrated to a paste-like system up to 20 mg mL^{-1} via static evaporation at 100°C. Before any sample loading and testing, the paste was gently stirred with a magnet stirrer at a constant rotational speed of 100 min^{-1} for 10 min. All the described sample preparation steps were carried out at room temperature, otherwise stated.

c. Characterization of Materials

<u>Transmission electron microscopy</u>: Transmission electron microscopy images were taken with a JEM-1400 (JEOL) transmission electron microscope, operating at 100 kV. The samples were prepared by drop casting HRCM dispersions onto ultrathin C-film onto holey carbon 400 mesh Cu grids (Ted Pella Inc.). The HRCM dispersion was diluted 1:50 before their deposition. The grids were stored under vacuum at room temperature to remove the solvent residuals.

<u>Raman spectroscopy</u>: Raman spectroscopy measurements were carried out by using a Renishaw microRaman inVia 1000 using a 100× objective (numerical aperture of 0.75), with an excitation wavelength of 532 nm and an incident power on the samples of 5 mW. The samples were prepared by drop casting the 1:30 diluted HRCM dispersion in IPA onto a Si wafer covered with 300 nm thermally grown SiO_2 (LDB Technologies Ltd.). The bulk graphite was analyzed

in the powder form. For each sample, 50 spectra were collected. OriginPro 2020 was used to perform the deconvolution and statistics.

Thermogravimetric analysis: Characterization of the thermal response of the conductive paste, and of its components, was performed by thermogravimetric analysis (TGA), using a simultaneous thermal DSC-TGA analyser (SDT-Q600, TA Instruments). The samples were heated from room temperature to 500 °C, with a constant ramp of 10 °C min^{-1}. Nitrogen, with a volume flow rate of 50 mL min^{-1}, was used as purge gas. The analysis was carried out for the conductive paste at the initial concentration of 10 mg mL^{-1}, after concentrating to 20 mg mL^{-1}, and after the final annealing treatment of the printed sheets, collecting the paste by mechanical removal from the sheet surface. In addition, the filler, the binder, and the solvent alone were analyzed, for comparison purposes. For each run, the weight *vs*. temperature was collected, and the first derivative curve (DTG) was calculated in order to highlight the temperatures corresponding to the highest weight change rates.

Rheology: Steady and dynamic simple shear measurements were carried out on conductive pastes on an MCR 102 rheometer in strain-controlled mode by Anton Paar equipped with Peltier temperature control system having a resolution of ±0.1 °C, and a Peltier hood as solvent trap connected to an external bath circulator in order to guarantee a uniform temperature gradient within the tested sample. Stainless steel parallel plates with 50 mm diameter were used as measuring fixture while setting the gap between plates equal to 1 mm as sample loading position. All the measurements were carried out on fresh sample, which was loaded at room temperature with the help of a metallic spatula. In order to avoid sample under filling issues, proper sample loading was always verified by eye-check prior testing. A small amount of low viscosity silicone oil (viscosity 49.3 mPa s at 25 °C, Alfa Aesar) is added at the free edges of the sample

in order to minimize solvent evaporation during measurements of twenty minutes and longer. All the measurements were performed at 20 °C with a delay time of 5 min at the set-up temperature in order to achieve thermal equilibrium of the sample. In particular, measurements of steady shear viscosity were performed by applying shear rates as a logarithmic ramp with values between 0.01 s^{-1} to 1000 s^{-1}. In order to assess the role of filler particles orientation on rheological properties of the conductive paste, different pre-shear values were applied before carrying out flow curve measurements. Moreover, back-and-forth viscosity ramps were also performed right after the initial flow curve to evaluate qualitatively the effect of flow deformation history on filler structure network rearrangement within the paste system.

d. Dipole Fabrication

Dipole antennas were implemented by deploying the HRCM-based paste on a cardboard foil substrate, having an approximate thickness of 0.5 mm, with relative dielectric permittivity ε_r = 1.8 and a loss tangent of 0.02.[2] The dipoles have half-lengths of 5 cm and 7 cm, respectively. The conductive paste was applied to the cardboard substrate through a screen printing process using a VEVOR (IT) Screen Printing Press machine using a frame with a mesh count of 110 per inch (equivalent to 220 µm fiber spacing) and a sieve opening of 45 %. Conductive paste sheets were prepared by spreading the paste on the frame with a spatula with rounded edges at a low enough rate. All printed paste sheets were annealed in a furnace at the temperature of 100 °C for 8 h, and then compressed on a hot plate at 300 °C for ∼ 15 s to 20 s in order to achieve a partial decomposition of EC and a much denser and more continuous network of HRCM flakes for higher electrical conductivity.

e. Simulation of Dipoles

The numerical model of the carbon-based pastes dipole antennas was developed in CST Microwave Studio 2020 (3DS, Germany). CST is a high-performance 3D full-wave commercial numerical software for electromagnetic components design, analysis and optimization. In this work we simulated the dipoles using the Time Domain Solver based on the Finite Integration Technique (FIT). FIT employs Yee-type cells on a cartesian volume mesh to discretize Maxwell's equations. [3] The equations are solved in an integral form, in a simple and efficient way, reducing computational costs and saving time. [3] The simulations settings are as follows: general purpose broadband sweep, tetrahedral mesh, adaptive mesh refinement min. 3, max. 8 passes with a 0.02 tolerance criterion of all the absolute values of S-parameters.

f. Characterization of Dipoles

The printed pattern resistivity was measured in a four-probe configuration by a RM3000 Test Unit (Jandel Engineering) equipped with a cylindrical probe having 4 in line electrodes spaced 1mm apart.

The return loss (RL) is a figure of merit that measure the reflected wave or signal strength traveling or being reflected to a transmitter or a source from an antenna. The reflected signal is the magnitude of the reflection coefficient S_{11}. In mathematical terms, return loss is defined as: [4]

$$RL = 20 \cdot \log_{10}(|S_{11}|^2). \qquad \text{(ES1)}$$

If $|S_{11}| \to 0, RL \to -\infty$ and the reflected signal is very low, thus implying a good matching of the antenna system with the reference impedance, in this case 50 Ω. The return loss was measured using a Hewlett-Packard 8720C VNA. An open, short, load (OSL) calibration

procedure was performed. A LPRS Straight 50 Ω SMA connector, welded to the terminal with the CircuitWorks silver-Epoxy conductive paste, has been used to connect and excite the dipole. A fundamental property of any antenna is its radiation pattern, which is the spatial distribution of a quantity which characterizes the electromagnetic field radiated by an antenna. [5] The normalized far-field pattern function U was measured as: [6]

$$U(\theta,\phi) \simeq \frac{1}{2Z_0}\left[|F_\theta(\theta,\phi)|^2 + \left|F_\phi(\theta,\phi)\right|^2\right] \quad \text{(ES2)}$$

where Z_0 is the characteristic impedance of the antenna, $F_\theta(\theta,\varphi)$ and $F_\phi(\theta,\varphi)$ are, respectively, the θ component of the far-field pattern function and the φ component of the far-field pattern function. The radiated power P_r was then derived as: [6]

$$P_r \simeq \frac{2\pi^2}{NM}\sum_{n=0}^{N-1}\sum_{m=0}^{M-1} U(\theta_n,\phi_m)\sin\theta_n \quad \text{(ES3)}$$

being N is the number of sample points along the θ-axis and M is the number of sample points along the φ-axis. [6] The antenna radiation pattern was measured within an anechoic chamber with dimensions of 8.0 m × 5.4 m × 3.6 m, as shown in **Figure S1**. We assumed, as customary, a spherical coordinate system (r,θ,ϕ), defined with respect to the antenna under test (AUT). The AUT was configured to receive signals using an antenna positioning system placed in the far-field zone of the transmitting antenna, specifically a double-ridged waveguide horn antenna (operating in the frequency range of 800 MHz to 18 GHz). When performing the measurement, we took care that the distance r between the horn and the AUT is much larger than the wavelength (λ), i.e., that the electrical condition $r \gg \lambda/2\pi$ is satisfied. [6] Furthermore, measures were performed in the case of distances much larger than the largest linear dimension of the antenna D. [6] Therefore, the geometrical condition $r \gg D$ is fulfilled. To reduce the phase

error, the Rayleigh condition $r>2(D/\lambda)^2$ was considered. [6] The received power has been measured through the transmission parameter provided by a VNA (Rohde & Schwarz ZVA 40).

2. Additional characterization

a. Transmission Electron Microscopy

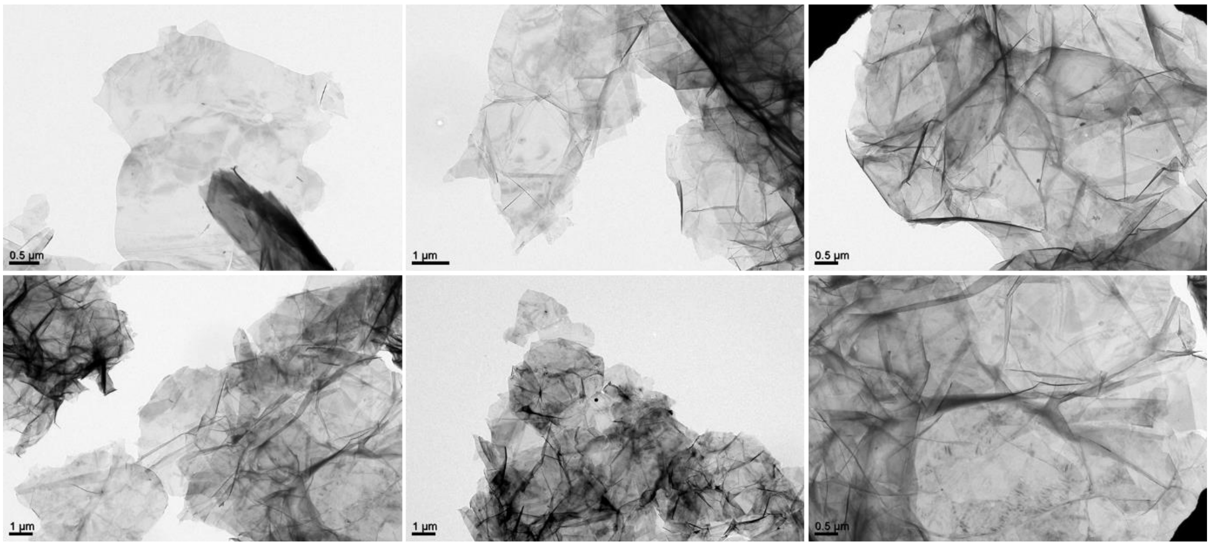

Figure S1: TEM micrographs displaying different parts of the HRCM material.

The transmission electron microscopy (TEM) analysis unveiled the presence of carbon material agglomerates, showcasing a flake-like morphology with a notable high aspect ratio. These agglomerates resulted in an average lateral size of 2.3 µm.

b. Raman Spectroscopy

The structural features of the as-produced HRCM were investigated using Raman spectroscopy. A peak at ∼1580 cm^{-1} corresponds to the E_{2g} Raman allows optical phonon, while a peak at ∼1350 cm^{-1} indicates an A_{1g} breathing mode, commonly seen in defected graphite. The

presence of these modes suggests HRCM affinity to graphene-like structures. Additional peaks at approximately 2700 cm^{-1} and ~2450 cm^{-1} were also observed, attributed to overtone vibrations. The statistical analysis conducted on the Raman spectroscopy data of the as-produced HRCM material involved an examination of the intensity ratio of the 2D peak components. The intensity ratio of the 2D peak components serves as an important indicator for estimating the average thickness of the material. This ratio is determined by comparing the intensities of different peaks within the Raman spectrum. Generally, a higher intensity ratio indicates a thinner material, while a lower ratio suggests a thicker one. In the case of HRCM, the statistical analysis of the Raman spectrum data revealed a higher intensity ratio of the 2D peak components. This implies that the material exhibits a relatively thin structure. Such a characteristic is indicative of similarities with graphene-like structures.

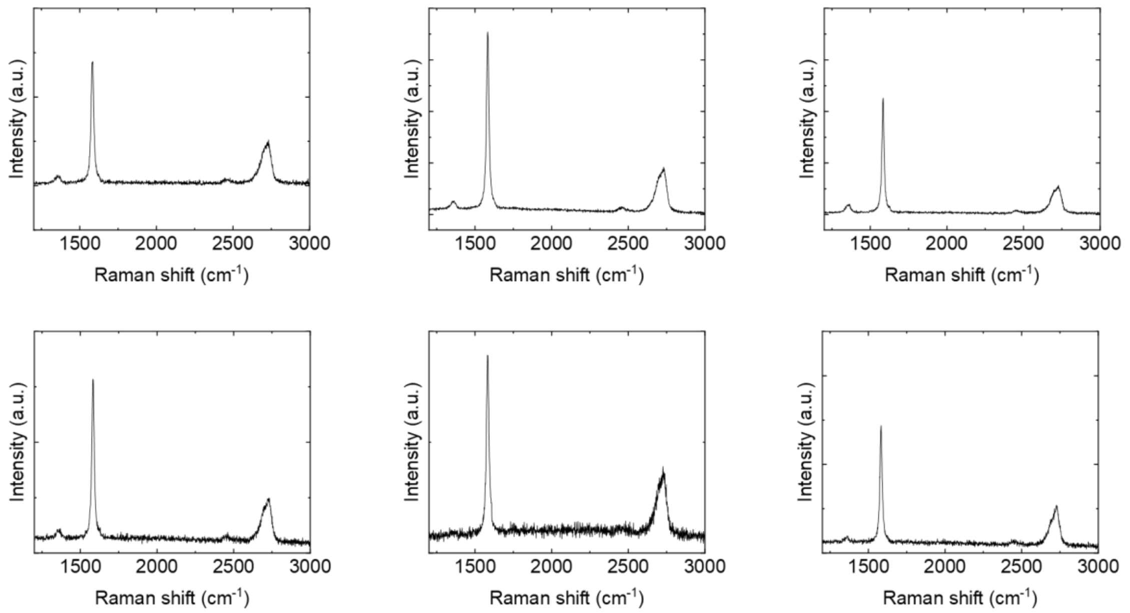

Figure S2: Different Raman spectra of the HRCM material.

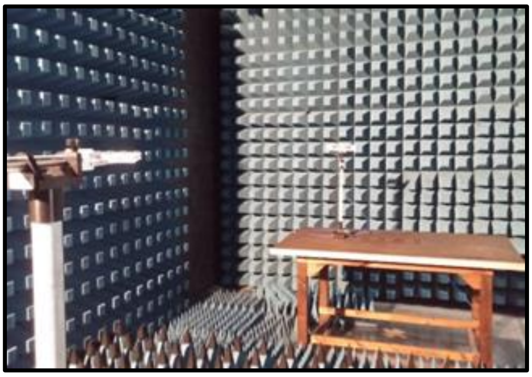

Figure S3: Characterization of the HRCM dipoles in the anechoic chamber.

[1] W. J. Hyun, E. B. Secor, M. C. Hersam, C. D. Frisbie, L. F. Francis, *Adv. Mater.* **2015**, *27*, 109.

[2] H. Saghlatoon, L. Sydänheimo, L. Ukkonen, M. Tentzeris, *IEEE Antennas Wirel. Propag. Lett.* **2014**, *13*, 915.

[3] Z. Chen, C.-F. Wang, W. J. R. Hoefer, *IEEE Trans. Microw. Theory Tech.* **2022**, *70*, 955.

[4] T. S. Bird, *IEEE Antennas Propag. Mag.* **2009**, *51*, 166.

[5] A. Roederer, E. Farr, L. J. Foged, M. Francis, R. Hansen, R. Haupt, K. Warnick, *IEEE Std* **2014**, 145.

[6] J. Fordham, L. Foged, V. Rodriguez, others, In *Antennas Propag. Standards Committee IEEE Antennas Propag. Soc.*, **2022**, p. 143.
